# In Regard to Kato et al

**DOI:** 10.1016/j.adro.2023.101366

**Published:** 2023-11-15

**Authors:** Claus Maximilian Bäcker, Christian Bäumer, Kevin Kröninger, Jörg Wulff, Beate Timmermann

**Affiliations:** a West German Proton Therapy Centre Essen, Essen, Germany; b West German Cancer Center, Essen, Germany; c University Hospital Essen, Essen, Germany; d Department of Physics, TU Dortmund University, Dortmund, Germany; e German Cancer Consortium (DKTK), Essen, Germany; f Department of Particle Therapy, University Hospital Essen, Essen, Germany

Dear editors,

With special interest, we read the recently published work by Kato et al.^1^ In general, we believe that a validation of the activation data from the commonly used databases is needed for any high-accuracy application in medical physics.

In the abstract, Kato et al^1^ outlined the goal of the study as to “identify the induced radionuclides produced from dental metals in proton beam therapy.” However, the radionuclides produced in metal implants can be taken from nuclear physics databases, similar to what was done for titanium in the past.^2^^,^^3^ In general, we appreciate that the authors were able to identify several radionuclides to confirm our work and add more of them, not detected due to the long delay between activation and measurement in a previous study.^3^ The authors commented on potential side-effects of radioactivation of the implants. In our opinion, this question can easily be answered by simple calculation with validated production cross sections. After a couple of days, the activity will reach the background level.^3^ Radioactivity at the environmental level should not have any effect on the clinical outcome.

Furthermore, the authors compared their measurements using Monte Carlo simulations, which of course were based on the reported cross sections. At this point, it might be more interesting to calculate activation cross sections from the activity. As the presented study featured a high-purity germanium detector, which facilitates a high accuracy level in terms of count rate and geometric efficiency,^4^ the calculation of cross sections may help to improve the reliability of simulations. In the past, the reliability of the cross-section data were questioned^5^^,^^6^^,^^7^ (and references therein), and thus, experimentally determined cross-section data are of high interest. Uncertainties of cross sections directly translate in uncertainties of Monte Carlo simulations.

In conclusion, we think there is need for more studies in the field of radioactivation by proton fields. These should address the sparse cross-sectional data and aim to confirm or even reduce the uncertainties.

## Disclosures

The authors declare that they have no known competing financial interests or personal relationships that could have appeared to influence the work reported in this paper.
